# An 8-gene predicting survival model of hepatocellular carcinoma (HCC) related to pyroptosis and cuproptosis

**DOI:** 10.1186/s41065-023-00288-7

**Published:** 2023-07-18

**Authors:** Hongjin Wang, Nian Wang, Ze Tang, Qiuyu Liu, Shiyu Nie, Wu Tao

**Affiliations:** Department of Critical Care Medicine, Yongchuan Hospital, Chongqing Medical University, Yong Chuan, Chongqing, 402160 China

**Keywords:** Hepatocellular carcinoma, Prognostic model, Pyroptosis, Cuproptosis, Bioinformatics

## Abstract

**Background:**

The study aimed to establish a prognostic survival model with 8 pyroptosis-and-cuproptosis-related genes to examine the prognostic effect in patients of hepatocellular carcinoma (HCC).

**Methods:**

We downloaded gene expression data and clinical information of HCC patients from The Cancer Genome Atlas (TCGA), International Cancer Genome Consortium (ICGC) and Gene Expression Omnibus (GEO). The clustering analysis and cox regression with LASSO were used for constructing an 8 PCmRNAs survival model. Using TCGA, ICGC and GEO cohort, the overall survival (OS) between high- and low- risk group was determined. We also evaluated independent prognostic indicators using univariate and multivariate analyses. The relatively bioinformatics analysis, including immune cell infiltration, function enrichment and drug sensitivity analyses, was performed as well. The gene expression of 8 PCmRNAs in vitro were validated in several HCC cell lines by qRT-PCR and Western blot. The relationship between *GZMA* and Fludarabine were further checked by CCK-8 assay.

**Results:**

The survival prognostic model was constructed with *ATP7A, GLS, CDKN2A, BAK1, CHMP4B, NLRP6, NOD1* and *GZMA* using data from TCGA cohort. The ICGC and GEO cohort were used for model validation. Receiver operating characteristic (ROC) curves showed a good survival prediction by this model. Risk scores had the highest predictable value for survival among Stage, Age, Gender and Grade. Most Immune cells and immune functions were decreased in high-risk group. Besides, function enrichment analyses showed that steroid metabolic process, hormone metabolic process, collagen − containing extracellular matrix, oxidoreductase activity and pyruvate metabolism were enriched. Potential drugs targeted different PCDEGs like Nelarabine, Dexamethasone and Fludarabine were found as well. *ATP7A, GLS, CDKN2A, BAK1, CHMP4B*, *NOD1* were upregulated while *NLRP6* and *GZMA* were downregulated in most HCC cell lines. The potential therapy of Fludarabine was demonstrated when *GZMA* was low expressed in Huh7 cell line.

**Conclusion:**

We constructed a novel 8-gene (*ATP7A, GLS, CDKN2A, BAK1, CHMP4B, NLRP6, NOD1* and *GZMA*) prognostic model and explored potential functional information and microenvironment of HCC, which might be worthy of clinical application. In addition, several potential chemotherapy drugs were screened and Fludarabine might be effective for HCC patients whose *GZMA* was low expressed.

**Supplementary Information:**

The online version contains supplementary material available at 10.1186/s41065-023-00288-7.

## Introduction

Hepatocellular carcinoma (HCC) was ranked as the fourth leading cause of cancer-related mortality and was gradually becoming a public health problem worldwide [1]. Previous studies had demonstrated that the incidence of HCC had increased in recent years [2, 3]. Despite of promising advances in treatment of HCC, the treatment of HCC remained ineffective and essentially palliative [4]. The unclear etiology of HCC made the disease extremely challenging to diagnose and treat. Therefore, identifying novel prognostic models could be helpful in accurately forecasting the overall survival (OS) of HCC patients [5].

Pyroptosis, also named as caspase-1-dependent cell death, was regarded as a new type of programmed cell death that caused the release of inflammatory mediators [6, 7]. It had emerged as a hotspot in cancer research in recent years [6]. The recent studies indicated that it was related to varieties of cancer diseases including gastric cancer, glioblastoma, breast cancer and etc. [8–10]. As for HCC, pyroptosis occurred in macrophages participated in triggering natural killer cell-mediated cytotoxicity to inhibit HCC progression [11]. Thus, we wondered whether overall survival of HCC patients was related to pyroptosis.

Cuprotosis was first identified in March 2022 as a novel form of cell death [12]. It had been proven that excess intracellular copper aggregated the lipoylated dihydrolipoamide S-acetyltransferase (DLAT), leading to toxic protein stress and, ultimately, cell death [13]. Copper also had been reported to have profound effects on tumorigenesis and played various regulatory functions in many cancer-related metabolic reprogramming, including fatty acid metabolism and glucose metabolism [14, 15]. Besides, some researchers noticed that copper metabolism could be a new biomarker for the detection of HCC metastasis as well as targeted therapy of HCC [16].

Pyroptosis and Cuprotosis were reported to have interactions on several disease. Copper exposure could induce microglia activation and lead it to secrete inflammatory products, resulting in the pyroptosis of dopaminergic neurons [17].Meanwhile, by generating ROS in hepatocytes, too much Cu could induce pyroptosis. And Cu-induced apoptosis could be attenuated after inhibition of Caspase-1-dependent pyroptosis as well [18].

The mRNA expression and clinical information of HCC were gathered for this research from TCGC and ICGC database to construct and validate the pyroptosis-and-cuproptosis-related prognostic model based on the differentially expressed genes. To investigate the potential mechanisms between HCC development and gene expression of pyroptosis and cuproptosis, functional enrichment analysis was carried out. In addition, different bioinformatic analyses, like immune status, tumor microenvironment, drug-sensitive analysis were performed to understand the pyroptosis-and-cuproptosis-related mRNA expression and its potential role in progression of HCC. The gene expression of 8 PCmRNAs were validated in Huh7, HepG2, Hep3B and HL7702 by qRT-PCR and Western blot. What’s more, the genes obtained in this scenario might be potential biomarkers and therapeutic targets for HCC. Here we also found Fludarabine might be the potential therapy drug for patients whose *GZMA* was low expressed.

## Method

### Datasets

The clinical information of HCC patients and corresponding cancer tissue RNA-seq data were retrieved from the TCGA (https://portal.gdc.cancer.gov/repository), ICGC (https://dcc.icgc.org) and GEO (https://www.ncbi.nlm.nih.gov/geo/) database. TCGA cohort was used for prognostic model construction while ICGC and GEO (GSE14520) were used for validation. A number of 70 pyroptosis and cuproptosis related genes were selected out according to references from previous systematic reviews prior to 2022 and they were presented in Supplementary 1 [12, 19–23].

### Identifying differentially expressed genes.

In order to identify pyroptosis and cuproptosis related differential expression genes (PCDEGs), screening standards were set as *pValue* < 0.05. We performed String (Protein–Protein Interaction Networks, V: 10.5) database (https://string-db.org/) on PCDEGs to obtain the interactions between proteins-targeted genes. The co-expression between PCDEGs were obtained with R packages of “reshape2” and “igraph”. Then, limma R package(“ConsensusClusterPlus”) was used to further identify 23 candidate PCDEGs with the screening standards of |log_2_FC|> 1 and FDR < 0.05 in the consensus clustering analysis. The code related to gene screening were shared in Supplementary 2.

### Construction and validation of the prognostic model

9 Prognostic associated PCDEGs were filtered out by performing the univariate cox regression analysis (*p* < 0.05) in TCGA cohort. Then, we performed a penalized shrunken regression method named the leaset absolute shrinkage and selection operator (Lasso) to avoid the problem of overfitting. Based on minimum criteria, the penalty parameter (λ) adjustment was determined through tenfold cross-validation. Based on the results of a cox regression analysis in TCGA cohort, 8 prognostic PCDEGs were identified and took part in constructing the prognostic model in ICGC and GEO cohort. Then, we calculated each patient’s risk score according to the formula below: risk score = esum(each gene’s expression × corresponding coefficient). Then, a model for cuproptosis-and-pyroptosis-related prognostic indicator was developed as follows: Risk score = ∑i = 1 N(Explg*Coef). Based on the median risk score, HCC samples could be distributed into two groups: low-risk(< median) and high-risk(≥ median).

### Prognostic implication

Overall survival analysis and the time-dependent receiver operating characteristic (ROC) curve analysis was performed using the R packages of “survival”, “survminer”, “survivalROC” or “timeROC”. Besides, the areas under the curve (AUC) at different years and clinical factors were calculated. With the survival package in R, we conducted multivariate and univariate independent prognostic analyses in TCGA and ICGC group, respectively.

### Clinical implications

The available clinical variables, such as age, gender, tumor grade and stage variables, were compared to assess the clinical implication of the model and depicted by R packages("ggpubr"). The CIBERSORT immune cell infiltration analyses were involved to count the infiltrating immune cells levels in HCC patients from TCGA. To predict the 1, 3, 5 years OS of HCC and improve the possibility for clinical usage, the nomogram prediction model was constructed based on 8 prognostic PCDEGs by using the “rms” R package. The calibration curves were plotted to assess the calibration of the nomogram.

### Immune status and tumor microenvironment (TME)

By using “gsva” R package, the levels of 16 types of immune cells were determined and 13 immune-related functions were computed via single-sample gene set enrichment analysis (ssGSEA). Two-way statistical ANOVA analysis was conducted here for assessing group difference in immune infiltration subtype in two groups. The relationship between tumor stemness, obtained from TCGA dataset, and risk score were performed by spearman correlation analysis. We evaluated the cell infiltration levels by calculating the immune and stromal scores with ESTIMATE algorithm.

### Functional enrichment analysis

Kyoto Encyclopedia of Genes and Genomes (KEGG) and Gene Ontology (GO) enrichment analyses of the PCDEGs were conducted by using R clusterProfiler package, respectively.

### Drug-sensitive analysis

We downloaded the transcriptional expression of NCI-60 human cancer cell lines from a publicly available dataset named CellMiner project page (https://discover.nci.nih.gov/cellminer) to determine the connection between diagnostic PCDEGs and drug sensitivity with the help of Pearson correlation analysis.

### Quantitative real-time PCR (qRT-PCR)

In this research, Huh7, HepG2, Hep3B and HL7702 were used to verify the expression of prognostic genes. The Hep3B, HepG2, Huh7 cell line (Human HCC cell) and HL7702(Human Normal Cells) were purchased from the Chinese Academy of Medical Sciences (Beijing, China). The total RNA was isolated by Trizol reagent (Life Technologies Corporation, Carlsbad, CA, USA). Next, using Superscript II reverse transcriptase, the synthesis of 20 μL cDNA was derived from 0.8 μg mRNA. QRT-PCR was performed on an ABIPRISM 7300 Sequence Detection System with SYBR Green PCR Master Mix (Applied Biosystems). The primers used in this study were *ATP7A(* forward 5’- TGACCCTAAACTACAGACTCCAA -3’, reverse 5’- CGCCGTAACAGTCAGAAACAA -3’*), GLS1(* forward 5’- AGTTGCTGGGGGCATTCTTTTAGTT -3’, reverse 5’- CCTTTGATCACCACCTTCTCTTCGA -3’*), CDKN2A(* forward 5’- ATGTCGCACGGTACCTG -3’, reverse 5’- GTTGTGGCCCTGTAGGA -3’*), BAK1(* forward 5′- GTTTTCCGCAGCTACGTTTTT -3′, reverse 5′- GGGACTCGTCTTCAGGGGAA -3′*), CHMP4B*(forward 5′- AGAACATGGGCTATGCCGCC—3′, reverse 5′- GCTCATCCTCGTCAAACTCTTCTCC -3′)*, NLRP6* (forward 5′-TTCGGCTGCATGGTTTCAGAG − 3′, reverse 5′-CGTCTCGTACAGGCAGTACAG − 3′)*, NOD1* (forward 5′- TACTGAAAAGCAATCGGGAACT -3′, reverse 5′-GTAGAGGAAGAACTCGGACACC -3′) and *GZMA*(forward 5′- AGTGCATCTTGGTCCGATACTC -3′, reverse 5′- GCTTCCAGAATCTCCATTGCAC -3′).

### Transfect

Vectors expressing *GZMA* were designed and synthesized by Sangon (Shanghai, China). HCC cell line of Huh7 was cultivated overnight to reach 70%–80% confluence. We transfected vectors at a dose of 10 nM by Lipofectamine 3000 reagent (Thermo Fisher Scientific). Negative control (NC) cells were empty vector-transfected cells. Control (C) cells were untransfected cells.

### Cell counting Kit-8 ( CCK-8 assay)

CCK-8 assay was used to measure the viability of Huh7 and HL7702 under treatment of Fludarabine ( Med Chem Express, Shanghai, China). The cells were resuspended and seeded in a 96-well plate (6 × 10^4^ cells/well) and cultured at environment of 37 °C. Each well was added with 10ul cck-8 solution (Yeasen, Shanghai, China) and the proliferation ability were represented with the absorbance at 450 nm tested by Microplate Reader (Bio-Rad, Hercules, CA).

### Western blot

The whole cell lysates (Beyotime, Shanghai, China) were used to harvest total protein. The equal amount of protein extract was then separated by SDS-PAGE and transferred electrophoretically to polyvinylidene difluoride membrane. Subsequently, membranes were blocked in 5% non-fat dry milk. The membranes were incubated with the appropriate dilutions of primary antibodies against *NOD1* antibody (1:1000, ab217798, Abcam, Cambridge, MA, US), *BAK1* (1:1000, ab32371, Abcam, Cambridge, MA, US), *NLRP6* (1:1000, ab58705, Abcam, Cambridge, MA, US), and *GAPDH* (1;1500; Abcam, Cambridge, MA, USA) antibody sampler kit (9782 T) at 4 °C. The more detailed steps have been illustrated as previously described [24].

## Results

### Differential expression of PCDEGs between the HCC and normal tissue.

**Fig. 1:**
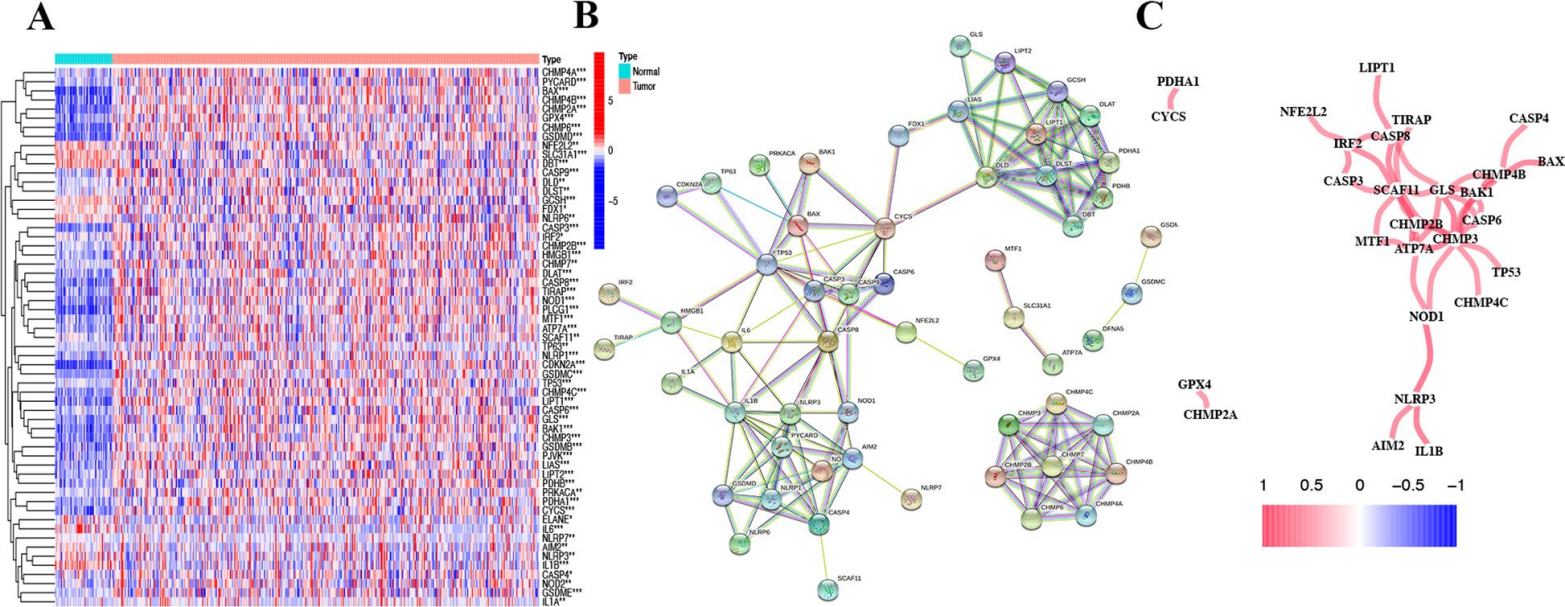
Identification of PCDEGs by array screening. **A** Heatmap of the PCDEGs between tumor group and normal group. blue: low expression level; red: high expression level (**B**) Protein–protein interaction network between these genes(cutoff = 0.5). **C** The co-expression between these genes(cutoff = 0.5). ***p < 0.001, **p < 0.01, *p < 0.05

Fifty normal samples and 374 tumor samples were obtained from TCGA to participate in the identification of candidate pyroptosis and cuproptosis related differential expression genes (PCDEGs). The clinicopathological findings of HCC patients in TCGA were extracted as well. 59 PCDEGs were identified and expressed differentially between normal and HCC patients (Fig. 1A). The protein–protein interaction (PPI) network and co-expression of these genes showed that the genes could be roughly divided into three categories, which were GSDM family regulatory network, pyruvate dehydrogenase and caspase family regulatory network (Fig. 1B-C).

### Tumor Classification in TCGA

**Fig. 2:**
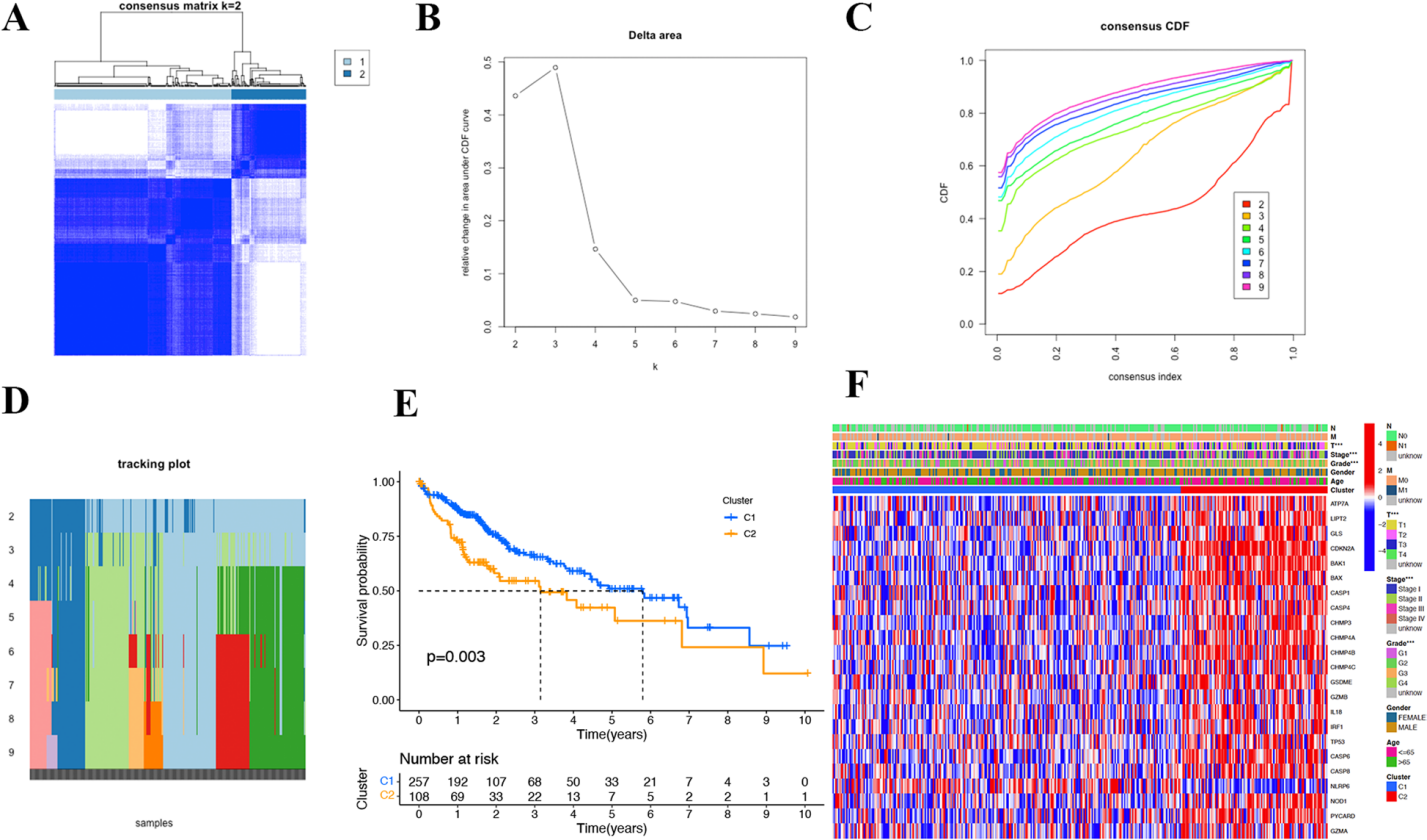
Identification of prognostic PCDEGs by gene cluster. **A** Two different gene clusters(k = 2). **B** Consensus clustering CDF of K from 2 to 10. **C** Relative change in area under the CDF curve of k from 2 to 9. **D** CDF tracking plot with examples from k = 2 to k = 9. **E** Survival probability between two gene clusters. **F** Heatmap of the gene expression of two gene clusters. ***p < 0.001

In accordance with the consensus clustering matrix, HCC patients obtained from TCGA were divided into two clusters. According to the cumulative distribution function (CDF) value, we found it was when k = 2 that we observed the highest intragroup correlation and low intergroup correlation (Fig. 2A-D). We then compared the OS of the two clusters and found the patients from cluster 1 generally had longer survival time than those from cluster 2 (*p* = 0.003), which indicated a vital prognostic value of these PCDEGs (Fig. 2E). The 23 candidate PCDEGs were further screened from the PCDEGs obtained above based on the result of the clustering. Here we noticed the two groups showed no differences in clinical parameters such as gender, age, M and N, while they differed in T, stage and Grade ( *p* < 0.001) (Fig. 2F).

### The construction and validation of 8 PCmRNAs prognostic model in TCGA and ICGC

**Fig. 3:**
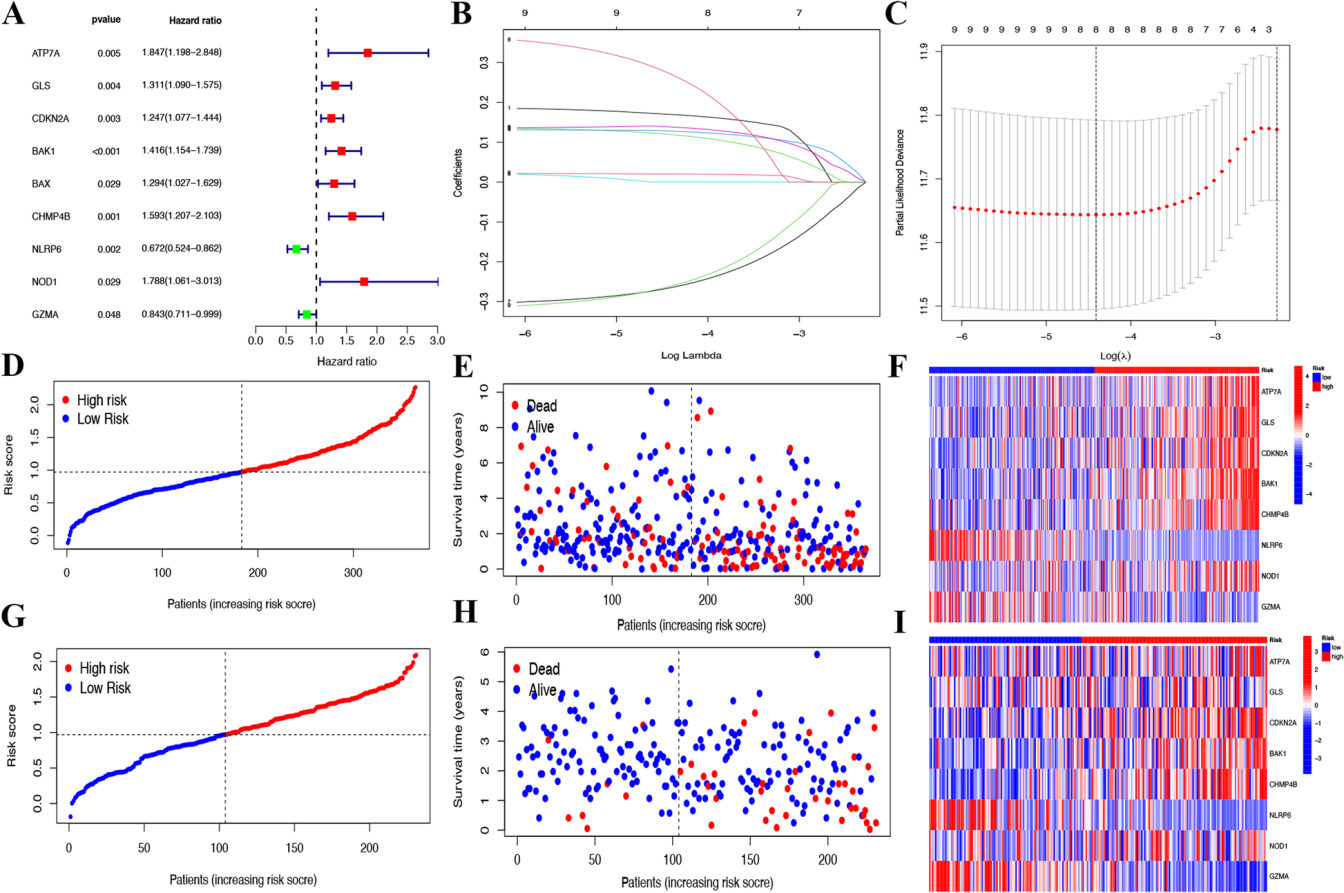
Construction and validation of 8 PCmRNAs prognostic signature. **A** A univariate Cox regression analysis in prognostic PCDEGs. **B**-**C** Partial likelihood deviance for different numbers of variables by Lasso Cox regression analysis. The median value and distribution of the risk scores in (**D**)TCGA and (**E**) ICGC datasets. The distribution of OS status in (**F**) TCGA and (**G**) ICGC datasets. The heatmap of the 8 prognostic PCmRNAs in (H)TCGA and (I)ICGC datasets

We first used univariate cox regression in 23 candidate PCDEGs and obtained 9 relative PCDEGs (Fig. 3A). Then LASSO cox regression was performed in 9 relative PCDEGs to identify the pyroptosis and cuproptosis related mRNAs (PCmRNAs) that played an essential role in model construction (Fig. 3B-C). Here we obtained 8 PCmRNAs (*ATP7A, GLS, CDKN2A, BAK1, CHMP4B, NLRP6, NOD1* and *GZMA*) for risk formula construction: 0.169897172150432 * *ATP7A* + 0.0203137804421373 * *GLS* + 0.119535844229867 * *CDKN2A* + 0.128136133840001 * *BAK1* + 0.138461460287378 * *CHMP4B* + -0.265097253909826 * *NLRP6* + 0.271551570082023 * *NOD1* + -0.261433885704348 * *GZMA.* The formula was used to compute the risk score of each patient. Median risk scores of TCGA and ICGC datasets across samples were applied as a threshold for dividing into two risk groups (Fig. 3D-E). Meanwhile, the scatter charts indicated that patients in high-risk groups might have a worse outcome in TCGA and ICGC datasets (Fig. 3F-G). The heatmap analysis implied that high-risk patients had increased expression levels of *ATP7A, GLS, CDKN2A, BAK1, CHMP4B* and *NOD1,* while *NLRP6 and GZMA* were more relevant to low-risk patients (Fig. 3H-I).

### Prognostic implication of the 8 PCmRNAs prognostic model

**Fig. 4:**
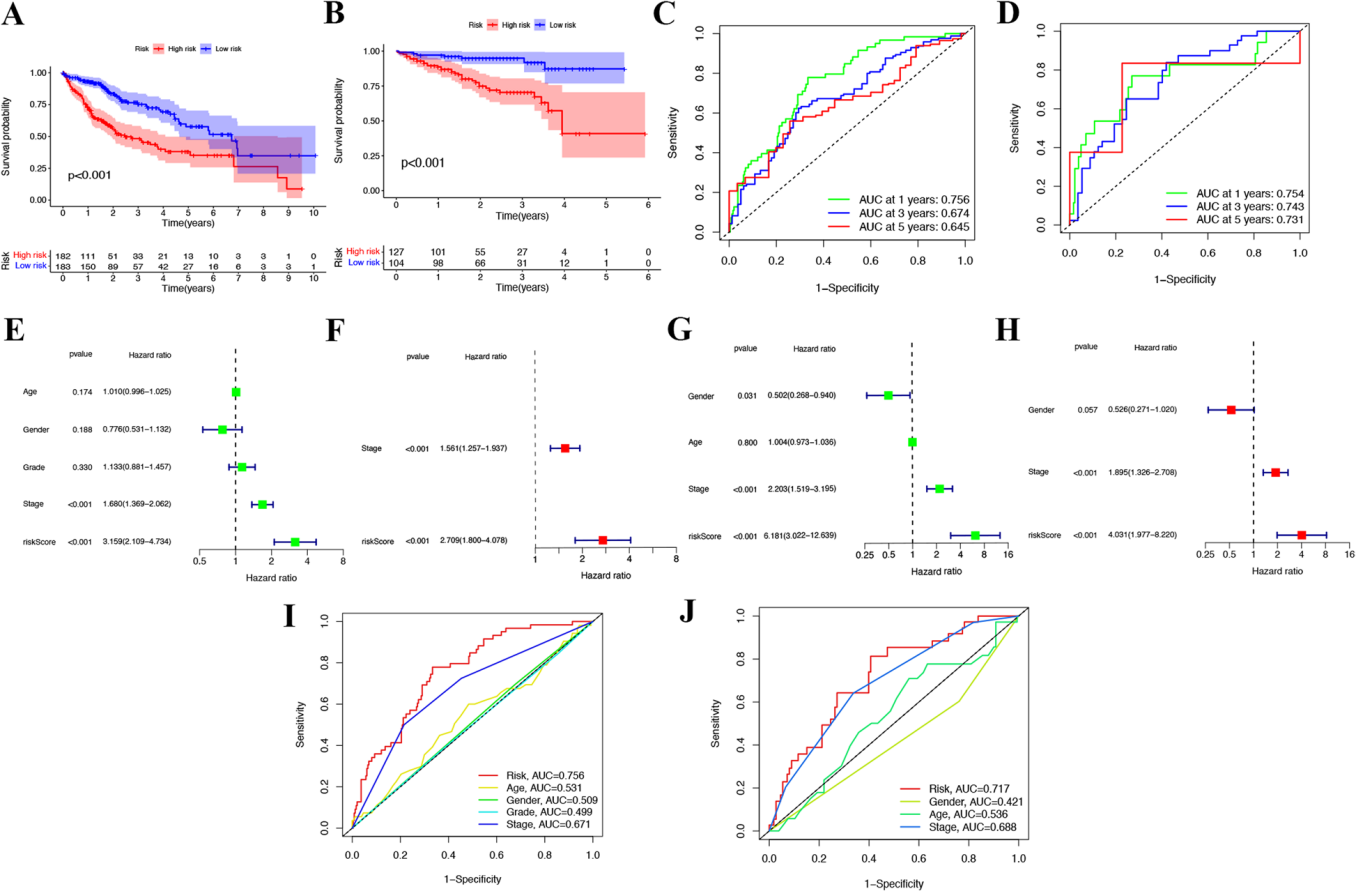
The prognostic role of the risk scores in the 8 PCmRNAs prognostic signature. KM curves of OS for patients in two risk groups of (**A**)TCGA and (**B**) ICGC datasets. Time-dependent OS ROC in (**C**)TCGA and (**D**) ICGC datasets. Univariate Cox regression for OS-related factors in (**E**) TCGA and (**F**) ICGC datasets. Multivariate Cox regression for OS-related factors in (**G**) TCGA and (**H**) ICGC datasets. Time-dependent ROC of OS for different clinical features in (**I**) TCGA and (**J**) ICGC datasets

The Kaplan–Meier (KM) curve demonstrated that the patients of the low-risk group had longer OS in TCGA datasets ( *p* < 0.001), which was further validated in ICGC datasets( *p* < 0.001) and GEO datasets ( *p* = 0.029) (Fig. 4A-B, Supplementary 3A). And ROC analysis for OS found that AUC was 0.756, 0.674, and 0.645 at 1, 3 and 5 respectively in TCGA (Fig. 4C). The Fig. 4D also demonstrated that the model was reliable for AUC was 0.754, 0.743, and 0.731 at 1, 3 and 5 respectively in ICGC. As for GEO dataset, the AUC was 0.608, 0.645, and 0.596 at 1, 3 and 5 respectively (Supplementary 3B). The risk score showed significance in both univariate Cox regression and multivariate Cox regression., indicating the risk score could be a significant variable for predicting prognosis in TCGA (HR = 2.709; CI = 1.800–4.078; *p* < 0.001) (Fig. 4E-F). We also validated it in ICGC(HR = 4.031; CI = 1.977–8.220; *p* < 0.001) (Fig. 4G-H) and GEO dateset (HR = 2.446; CI = 1.373–4.360; *p* = 0.002) (Supplementary 3C-D), and found the similar outcome. Among other traditional features, including Stage, Age, Gender and Grade, the AUC of the Risk had the highest predictable value (TCGA: 0.756; ICGC: 0.717) (Fig. 4I-J). Besides, we also validated predictable value of the prognostic model in GEO dataset (Risk = 0.653)( Supplementary 3E).

### Clinical implications of the 8 PCmRNAs prognostic model

**Fig. 5:**
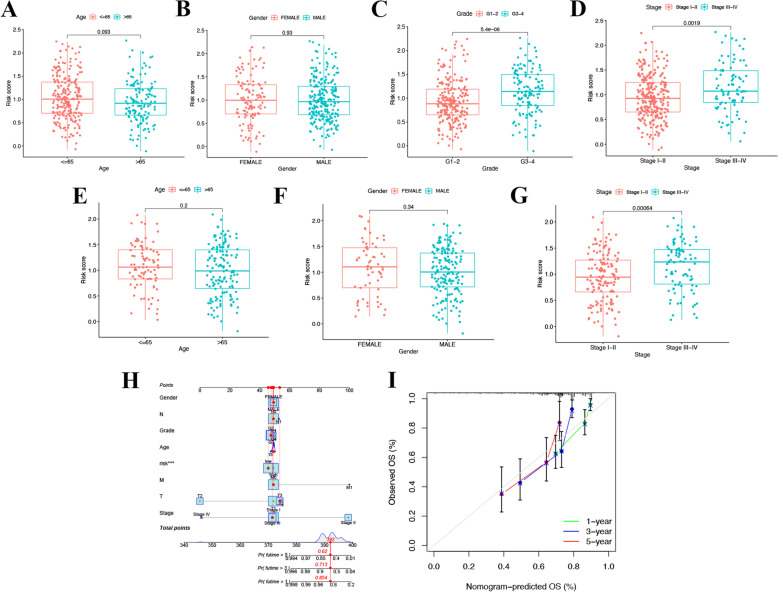
The role of risk score in various clinical characteristics and nomogram construction. In TCGA: (**A**) Age (**B**) gender, (**C**) grade and (**D**) stage. In ICGC: (**E**) Age (**F**) gender and (**G**) stage. **H** The nomogram for predicting HCC patients’ survival. (**I**) Calibration plots applied for predicting the 1-, 3- and 5-year OS in TCGA

For each dataset, we included clinical variables such as age, gender, grade and stage variables as explanatory variables to better evaluate the clinical implications of 8 PCmRNAs prognostic model. Here we noticed that patients with high grade and stage III − IV were more likely to have a high-risk score in TCGA, and the similar trend occurred in stage III − IV in ICGC (Fig. 5C-D, G). However, no significant differences were observed in Age and Gender (Fig. 5A-B, E–F).

In order to predict the 1-year, 3-year, and 5-year OS of HCC patients, we constructed a nomogram for clinical usage. After drawing a vertical line from the total point to the survival prediction axis, each patient could be predicted the 1-, 3- and 5-year OS (Fig. 5H). A relatively precise ability to predict the OS of HCC patients at 1—year, 3—year, and 5—year was demonstrated in F ig. 5I.

### The analysis of immune status and TME

**Fig. 6:**
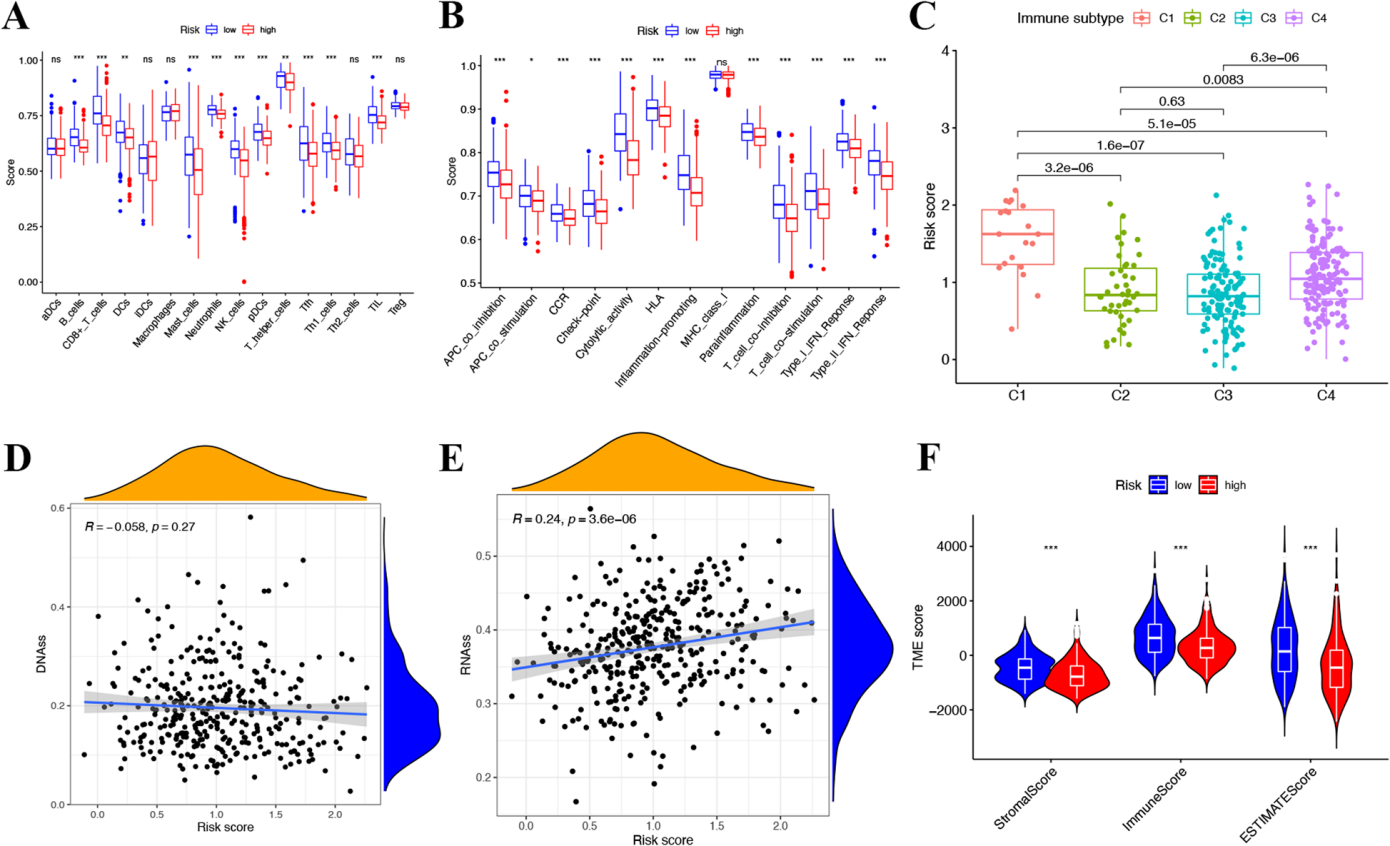
Immune status and tumor microenvironment analysis in the TCGA cohort. **A** The boxplots showed the scores of 16 immune cells. **B** The boxplots showed the scores of 13 immune-related functions. **C** Comparison of different immune infiltration subtypes based on different risk scores. The correlation between risk score and (**D**) DNAss and (**E**) RNAss. **F** The differential expression of Stromal, Immune, and Estimate Score for TME score

We compared the ssGSEA scores between different risk groups to evaluate the infiltration of the immune cells and explore immune functions. Here we found that there were significant enrichments of B cells, CD8 + T cells, dendritic cells (DCs), mast cells, neutrophils, natural killer cells (NK cells), plasmacytoid dendritic cells (pDCs) and etc. in low-risk group (*p* < 0.05) (Fig. 6A). Besides, CIBERSORT immune cell infiltration analyse was also perfomed to estimate the relative immune cell infiltration levels. Here we found B cells naïve, T cells CD8, T cells CD4 memory activated, T cells follicular helper and had the higher fraction in low-risk group, while Macrophages M0 and Macrophages M2 were enriched in high risk group (*p* < 0.05) (Supplementary 4). Furthermore, APC (antigen presenting cell) co-inhibiton and co-stimulation, chemokine receptors (CCR), check-point, cytolytic_activity, HLA (human leukocyte antigen), inflammation-promoting, parainflammation, Type I and Type II IFN responses, T cell co-inhibition and co-stimulation were selected out (Fig. 6B, *p* < 0.05).

Four types of immune infiltration types, including wound healing (C1), interferon gamma (IFN-γ) dominant (C2), inflammatory (C3), lymphocyte depleted (C4) were enrolled to detect the connection with signature. Here we observed infiltration of wound healing (C1) was highly associated with patients belong to the high-risk group, while interferon gamma (IFN-γ) dominant (C2), inflammatory (C3), lymphocyte depleted (C4) might happen in those with low-risk scores (Fig. 6C).

Next, we performed RNA stemness score (RNAss) and DNA methylation pattern (DNAss) to evaluate the tumor stemness. As presented in Fig. 6D-E, no significant correlation was observed between DNAss and Risk score, while a positive association was found between RNAss and Risk score (R = 0.24, *p* < 0.05). In addition, the risk score was significantly associated with the stromal score, immune score and estimate score of patients with HCC in low-risk group, demonstrating a more essential role of TME in low-risk group compared to high-risk group (Fig. 6F).

### Functional enrichment and pathway analyses

**Fig. 7:**
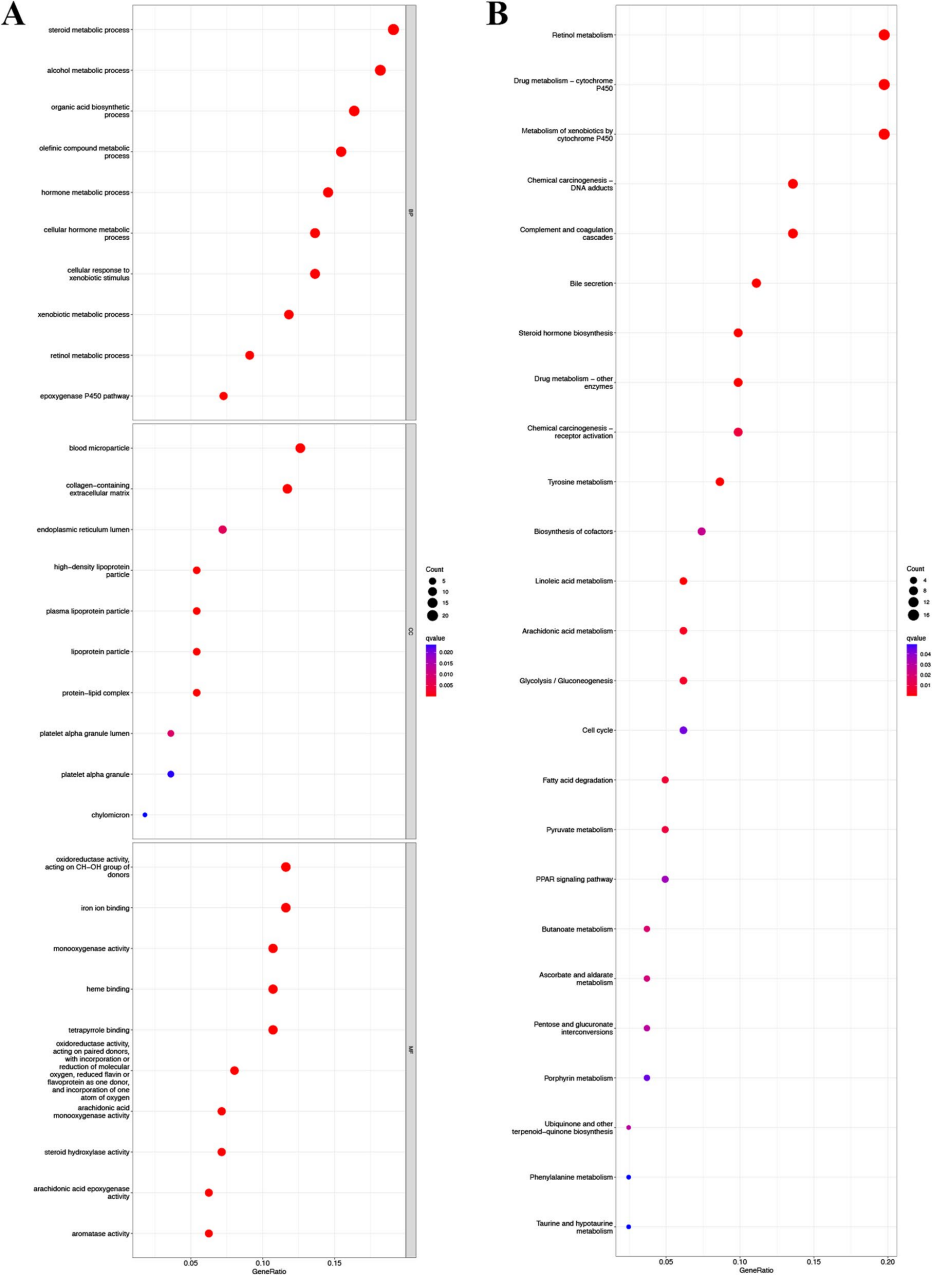
Functional analysis based on the 8 PCmRNAs between the two-risk groups in the TCGA cohort. **A** Bubble plot of GO enrichment. **B** Bubble plot of KEGG enrichment

We performed KEGG and GO function enrichment analyses based on risk classification. Three gene ontologies (GO) were selected: molecular function (MF), cellular component (CC), and biological process (BP). Expression analysis showed that PCDEGs were most enriched in biological processes like steroid metabolic process, hormone metabolic process, collagen − containing extracellular matrix, oxidoreductase activity and etc. (Fig. 7A). Furthermore, KEGG pathway enrichment analysis indicated that PCDEGs were significantly enriched in Drug metabolism–cytochrome P450, steroid hormone biosynthesis, pyruvate metabolism and etc. (Fig. 7B).

### The Chemotherapy Sensitivity of 8 PCmRNAs in TCGA

**Fig. 8:**
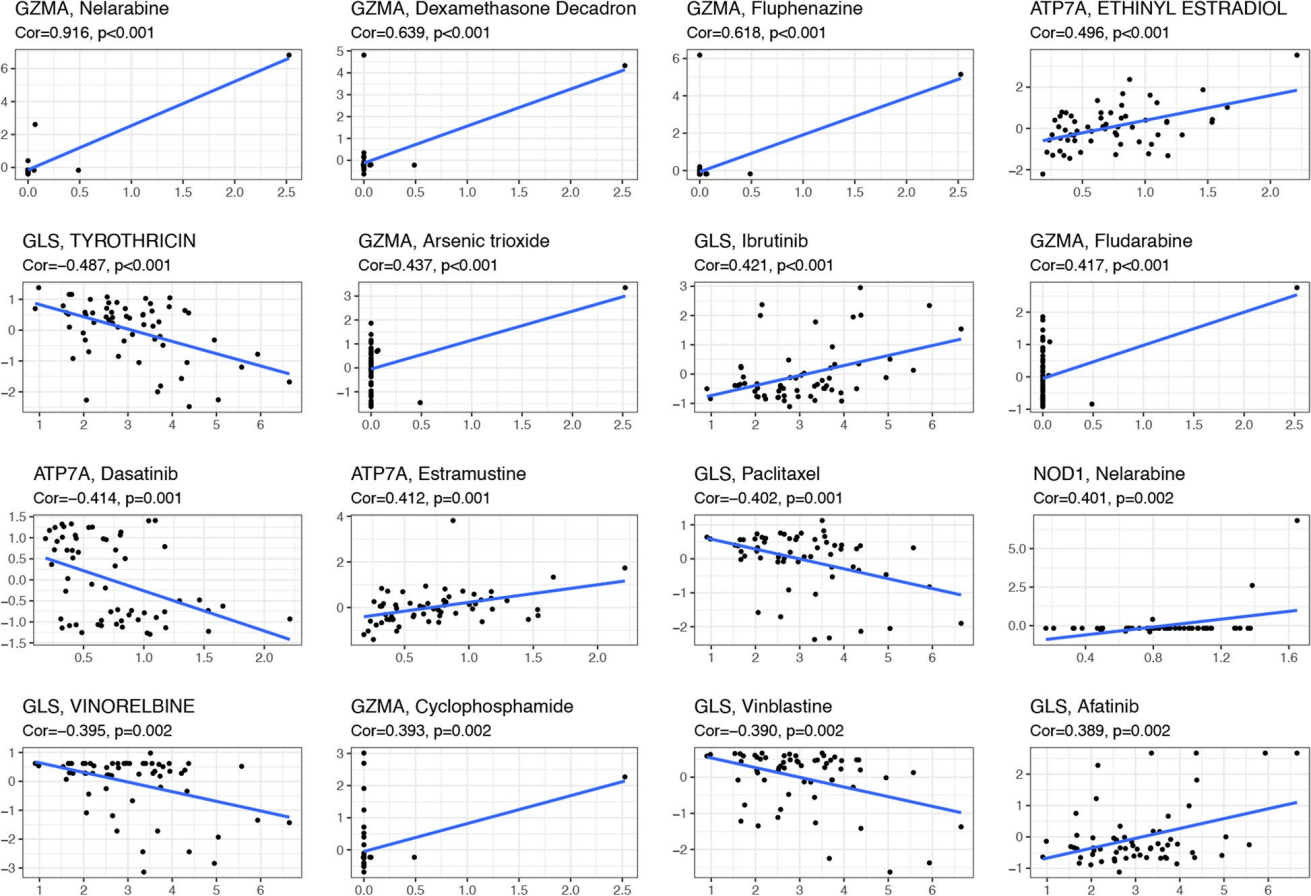
The scatter plots of top 16 correlation analsyses between PCmRNAs and drug sensitivity (X-axis: the levels of gene expression; Y-axis: the levels of IC50)

According to NCI-60, the top 16 correlation analyses between prognostic genes and drug sensitivity were chosen. Figure 8 demonstrated that the lower the expression of *GZMA*, the higher the sensitivity of Nelarabine, Dexamethasone, Fluphenazine, Arsenic trioxide, Fludarabine and Cyclophosphamide. *ATP7A* was negatively associated with drug sensitivity of ETHINYL ESTRADIOL and Estramustine, while it positively associated with drug sensitive of Dasatinib. Besides, the expression of *GLS* was negatively associated with drug sensitivity of Ibrutinib and Afatinib, while it was positively associated with TYROTHRICIN, Paclitaxel, VINORELBINE and Vinblastine. Moreover, *NOD1* was insensitive to Nelarabine (Fig. 8).

### The validation of 8 PCmRNAs in vitro

**Fig. 9:**
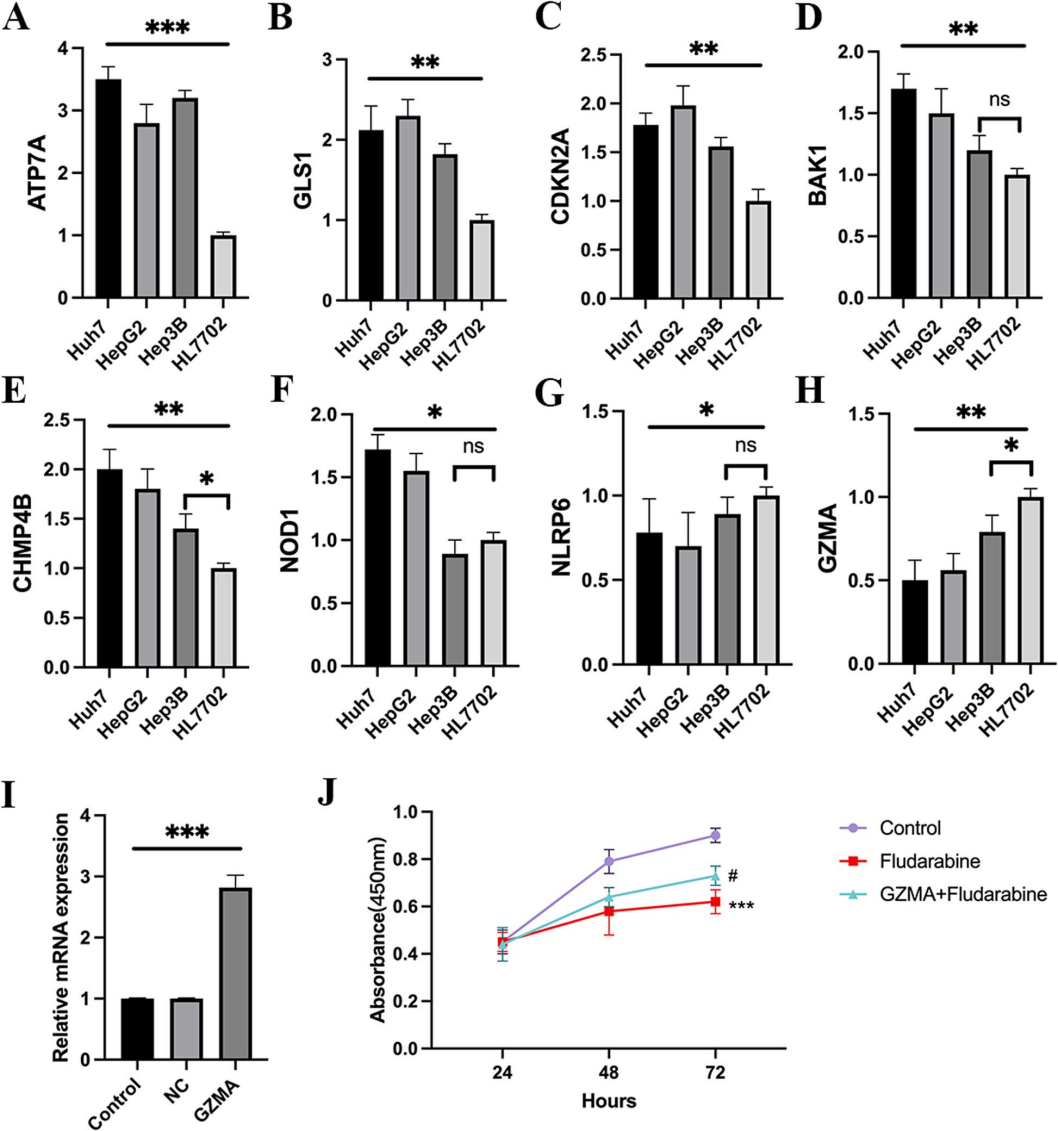
The validation of 8 PCmRNAs in vitro. **A**-**H** QRT-PCR of 8 PCmRNAs. *P < 0.05, **P < 0.01, ***P < 0.001, ns = no significant. **I** GZMA overexpression in HCC cell line of HuH7. **J** The cell viability of Hep3B under treatment of Fludarabine (5 μM). ***P < 0.001 (Fludarabine vs Con), ^#^*P* < 0.05 (GZMA + Fludarabine vs Fludarabine)

In this research, we also explored the gene expression in vitro in Huh7, HepG2, Hep3B and HL7702. Here we conducted the qRT-PCR on Huh7, HepG2, Hep3B and HL7702 and found the 5 PCmRNAs, including *ATP7A, GLS, CDKN2A, BAK1* and *CHMP4B,* were all upregulated in Huh7 and HepG2 compared to HL7702, although no significant difference was observed in *BAK1* and *NOD1* in Hep3B (Fig. 9A-F). While *NLRP6 and GZMA* were showing the opposite trends in Huh7 and HepG2, and no significant difference was observed in *NLRP6* in Hep3B (Fig. 9 G-H). For no significant differences were observed in *BAK1*, *NOD1* and *NLRP6*, we further validated the protein expression level of them in different cell lines by western blot. Although the similar results were obtained in Hep3B again except *BAK1*, we found the expression of *BAK1* and *NOD1* were highly expressed and *NLRP6* was low expressed in Huh7 and HepG2 ( Supplementary 5A-B).

Meanwhile, a kind of chemotherapy drug, Fludarabine, were introduced in to check the relationship between *GZMA* and anti-tumor effect of the potential drug obtained from this model. We over-expressed the *GZMA* and the overexpression of it was revealed by RT-qPCR in Huh7 (Fig. 9I). Here we noticed Fludarabine could significantly inhibit the cell viability of Huh7 in 72 h when *GZMA* was low expressed. When *GZMA* was overexpressed, the inhibition was reversed and a significant difference was observed between groups of Fludarabine and GZMA + Fludarabine (Fig. 9J).

## Discussion

HCC was one of the major factors of cancer-related deaths globally. The burden of HCC had been quickly rising due to the late diagnosis and restricted therapeutic choices [25, 26]. Up till now, orthotopic liver transplantation, surgical resection, local destruction, chemotherapy and application of biomaterials had been the most frequently used treatment options for HCC, while the results were still less than satisfactory [27–29].

Thus, a new prognostic model based on pyroptosis and cuprotosis was worthwhile to pursue for now. Though pyroptosis and cuprotosis were two different kinds of cell death, they had some common mechanisms on tumor progress. Too much Copper could induce pyroptosis in hepatocytes, and Cu-induced apoptosis could be attenuated after preventing Caspase-1-dependent pyroptosis from happening [18]. Copper-induced pyroptosis in jejunal epithelial cells via the IRE1α-XBP1 pathway was also reported recently [30]. To the best of our knowledge, no previous studies had ever examined the effects of pyroptosis-and-cuprotosis-ralated genes on development of HCC and explored their potential function information. In this study, we investigated the 59 PCDEG to depict the underlying molecular mechanism in the development of HCC tissue, constructed a novel 8- PCmRNAs prognostic model of HCC and reveled the 8 PCmRNAs’ related pathway and tumor microenvironment for the first time.

Firstly, 59 PCDEGs were differentially expressed between HCC and normal tissues, indicating a significant role of cuproptosis and pyroptosis in the prognosis of HCC. Here we noticed the genes could be divided into GSDM family regulatory network, pyruvate dehydrogenase and caspase family regulatory network. An integrated analysis of GSDMs in HCC showed that GSDM was most likely related to cell adhesion, growth regulation, and hormone metabolic process [31]. It was through down-regulation of pyruvate dehydrogenase kinase isozyme 1 (PDK1) mediated by the WNT/β-catenin pathway that PGC1α suppressed the HCC progression [32]. Caspase family, closely associated with apoptosis, was involved in the development of HCC through complicated pathway. For instance, Caspase 3 pathway inhibited the HCC progression under the regulation of lncRNA-PDPK2P [33].

Then, 8 prognostic PCmRNAs, including *ATP7A, GLS, CDKN2A, BAK1, CHMP4B, NLRP6, NOD1* and *GZMA* were screened after consensus clustering, univariate cox regression and LASSO cox regression. As a copper transporter gene, *ATP7A* was significantly altered in HCC samples and correlated with poorer survival in HCC patients [34]. *GLS* (glutaminase) promoted proliferation in HCC cells via AKT/GSK3β/CyclinD1 pathway [35]. We also noticed pyruvate dehydrogenase complex (PDHC) inhibition and *GLS* activation via MET-mediated phosphorylation promoted HCC metabolism and biogenesis [36]. *CDKN2A* was often regarded as a tumor suppressor and approximately 8% of HCC patients harbored *CDKN2A* deletions [37]. *BAK1* was an essential pathway involved in tumorigenesis of HCC, which made it a target with great therapeutic potential [38]. *CHMP4B* was interacted with Vps4A to decrease β-catenin signaling, and finally inhibited metastasis of HCC [39]. *NLRP6* exerted inhibitory effects on gastric cancer cell growth, which implicated the potential therapy application as well. [40, 41]. In recent study, up-regulated *NLRP6* was noticed to be related with intestinal candida albicans to promote hepatocarcinogenesis [42]. As for *NOD1* and *GZMA*, the former exerted its antitumor effect on HCC by inhibiting SRC-MAPK axis and the latter interacted with F2R to suppress the activation of HCC cells [43, 44]. Based on 8 PCmRNAs, a prognostic model was constructed and survival analysis showed that the signature worked well in predicting the OS of patients in the TCGA and ICGC cohorts. Every HCC patient’s risk score was obtained based on the expression of the 8 PCmRNAs in the prognostic signature, and the patients were divided into two groups: low-risk(< median) and high-risk based on median risk score. According to the signature and associated literature of the 8 PCmRNAs, we could say that the signature was closely relevant to HCC progression and therapy. What’s more, a nomogram with relatively precise predictive ability for predicting HCC was constructed and brought more possibilities to clinical application.

The presence of inflammation was always accompanied by pyroptosis and copper imbalance [45, 46]. Various studies had found chronic inflammation was involved in tumor initiation and promotion by suppressing the amounts or function of anti-tumor immune cells [47]. In this research, in the high-risk group, immune cells such as the B cells, CD8 + T cells, mast cells, neutrophils and NK cells, and immune pathways like APC co-inhibition and co-stimulation, CCR, and cytolytic_activity, were significantly decreased, which implied that decreased levels of anti-tumor immune cells or functions and increased levels of macrophages M2 contributed to cancer progression. These results also indicated that boosting anti-tumor immune responses was crucial for effective clinical therapies.

A detailed analysis of the immune infiltration of HCC was conducted to explore the role of risk score in immune infiltration. Here the results showed that the enrichment of C1(wound healing) mainly happened in patients belonging to high-risk group in analysis of microenvironment. Wound-healing response could participate in promoting the development of HCC, which was in accordance with our findings [48]. C2, C3, and C4 enrichment were more related to those with low-risk score, implying these types might be potential factors to inhibit the genesis and progression of HCC. Besides, significant differences were detected between high- and low-risk score with stromal score, immune score, estimate score and RNAss which again emphasized that TME might facilitate the development of HCC and RNAss could be an accomplice in it.

Meanwhile, the GO and KEGG analysis further depicted that PCDEGs significantly enriched in steroid metabolic process, hormone metabolic process, collagen − containing extracellular matrix, oxidoreductase activity, steroid hormone biosynthesis and pyruvate metabolism, which provided a glimpse into the involvement of pyroptosis and cuproptosis related mechanism in HCC development and progression. As far as we know, the regulation of mitochondrial cholesterol, including its role in mitochondrial routine performance, had been practically linked to initiation and progression of HCC [49]. The HCC microenvironment and cancer phenotype could be influenced after dysregulating of thyroid hormone signaling, raising the possibility that targeting thyroid hormone regulation might delay the progress of the HCC [50]. Abundant collagen produced by cancer-associated fibroblasts stimulated tumor progression by creating a favorable microenvironment for HCC [51]. Qikai Sun reported that xanthine oxidoreductase, which was expressed at low levels in human HCC, could block HCC propagation [52]. Genes expression of pyroptosis and cuproptosis filtered out these pathways, which meant targeting these PCDEGs and related pathways might be of significance in understanding the progression of HCC between patients in two risk groups.

Then we analyzed the data of 60 different cell lines to filter Food and Drug Administration (FDA)- approved chemotherapy drugs to enhance the gene-targeted drug sensitivity and avoid drug resistance. For instance, cancer cells were found to be more sensitive to THINYL ESTRADIOL and Estramustine with the increased expression of *ATP7A* genes, while they were insensitive to Dasatinib. In light of these findings, new precision strategy directions for drug treatment for HCC may be revealed in the future.

Finally, we checked the expression of the 8 genes in Huh7, HepG2, Hep3B and Hep3B. As for the expression of *GZMA* was higher in HCC cell lines compared to HL7702, and Fludarabine was suspected to be sensitive to HCC when low GZMA expression occurred in, we performed CCK-8 to compare the cell viability after exposure to Fludarabine or *GZMA* was highly expressed. The inhibition of Fludarabine, a kind of chemotherapy drug found by NCI-60 based on prognostic model, in Huh7 demonstrated the potential therapy of the drug especially when *GZMA* was low expressed. What’s more, Fludarabine might influence the biological pathway of collagen − containing extracellular matrix [53, 54]. Besides, to justify the clinical usefulness of the 8 genes, we provided nomogram predicting prognosis of a patient with HCC. The clinical prediction model established by nomogram, as well as the potential therapeutic drugs for these 8 genes discovered by bioinformatics and NCI-60, will help us diagnose and treat HCC patients. The main limitation of this research was the lack of a thorough mechanistic survey of relationship between the expression of *GZMA* and anti-tumor effect of Fludarabine.

## Conclusion

All in all, we constructed a novel prognostic model based on 8 PCmRNAs to predict the prognosis of HCC patients and explored related potential functional information and TME of the 8 PCmRNAs in HCC. The model and several potential chemotherapy drugs can provide useful insights into the clinical management of HCC.

## Supplementary Information

**Additional file 1. **70 pyroptosis-and-cuproptosis-related genes.

**Additional file 2.** The code related to gene screening.

**Additional file 3.** The predictive power of the risk model in GSE14520 cohort. ( A)KM curves of OS for patients in two risk groups. T ( B)ime-dependent OS ROC. Univariate Cox regression for ( C)OS-related factors and ( D) Multivariate Cox regression. ( E)Time-dependent ROC of OS for different clinical features.

**Additional file 4.** CIBERSORT immune cell infiltration analyses.

**Additional file 5. **( A)The protein expression of BAK1, NOD1 and NLRP6 in different cell lines by Western blot. ( B) The densitometric quantification levels of BAK1, NOD1 and NLRP6. **P < 0.01, ***P < 0.001, ns = no significant.

## Data Availability

The data sets supporting the results of this article are included within the article and its additional files.
