# Supplementary figures and images for: An 8-gene predicting survival model of hepatocellular carcinoma (HCC) related to pyroptosis and cuproptosis
Source: Hereditas. 2023 Jul 18;160:30. doi: 10.1186/s41065-023-00288-7 (PMC10353252; doi:10.1186/s41065-023-00288-7)

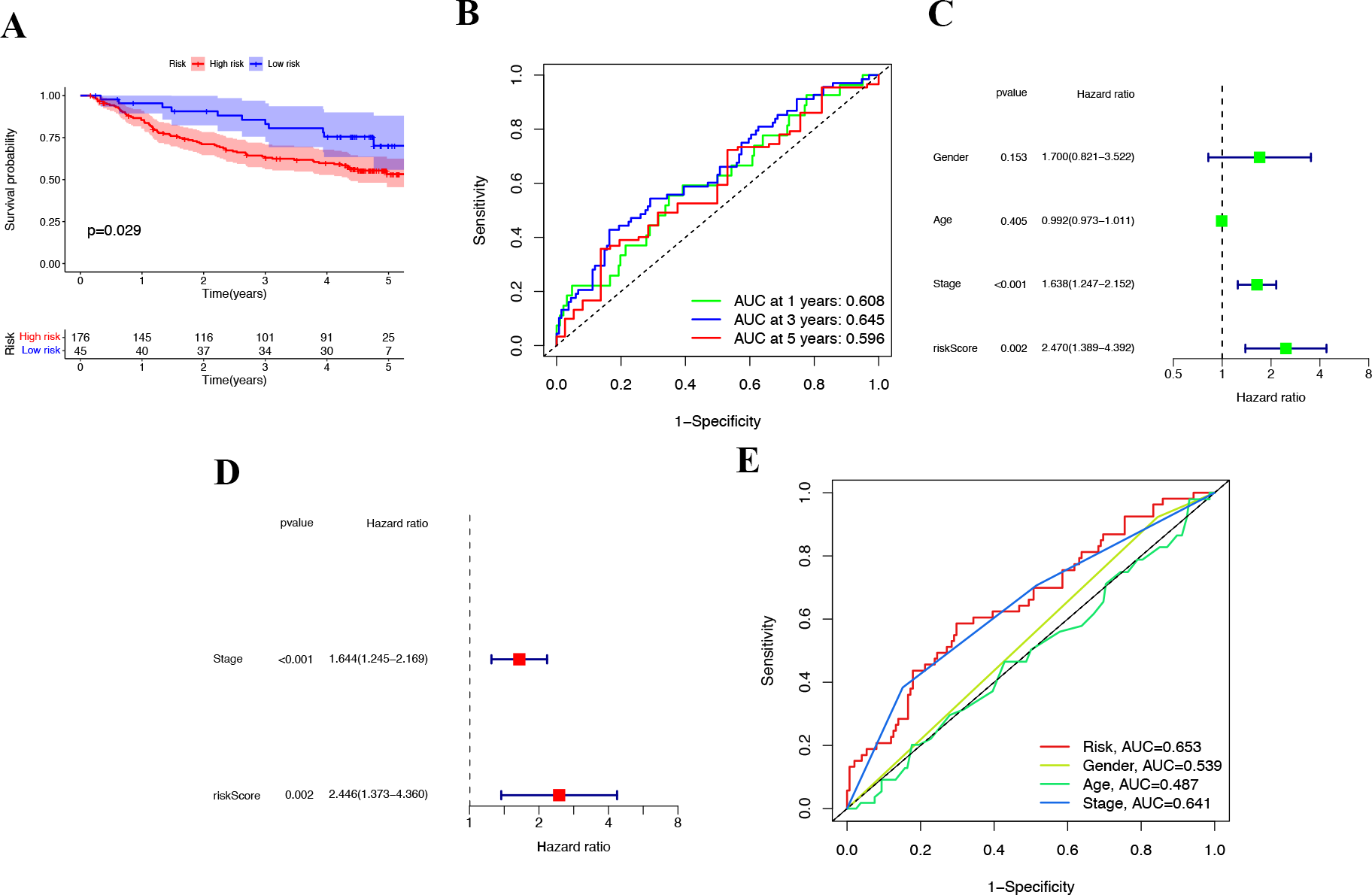

Supplement: Supplementary file 3 — Additional file 3. The predictive power of the risk model in GSE14520 cohort. ( A)KM curves of OS for patients in two risk groups. T ( B)ime-dependent OS ROC. Univariate Cox regression for ( C)OS-related factors and ( D) Multivariate Cox regression. ( E)Time-dependent ROC of OS for different clinical features. [file 41065_2023_288_MOESM3_ESM.tif]

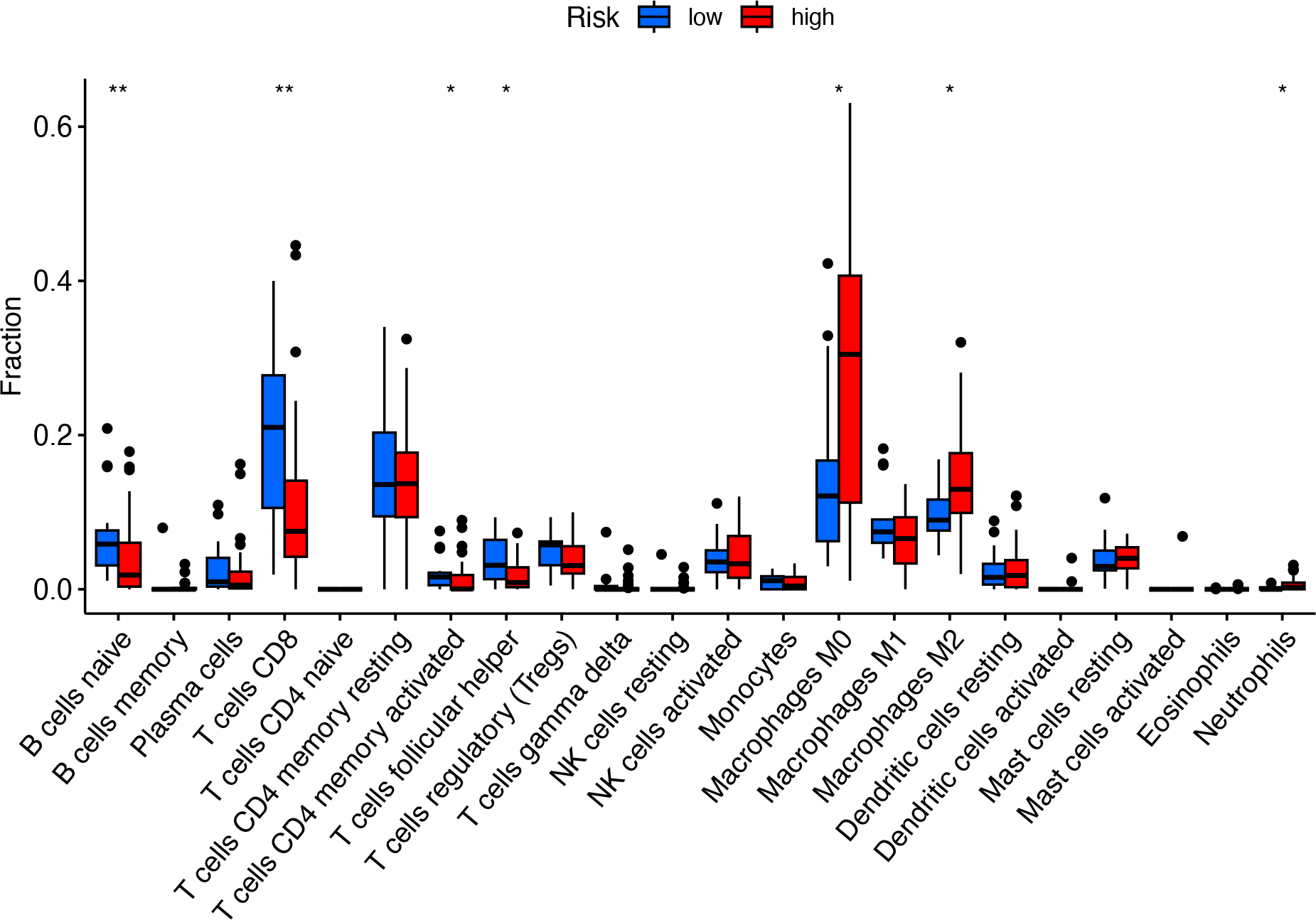

Supplement: Supplementary file 4 — Additional file 4. CIBERSORT immune cell infiltration analyses. [file 41065_2023_288_MOESM4_ESM.tif]

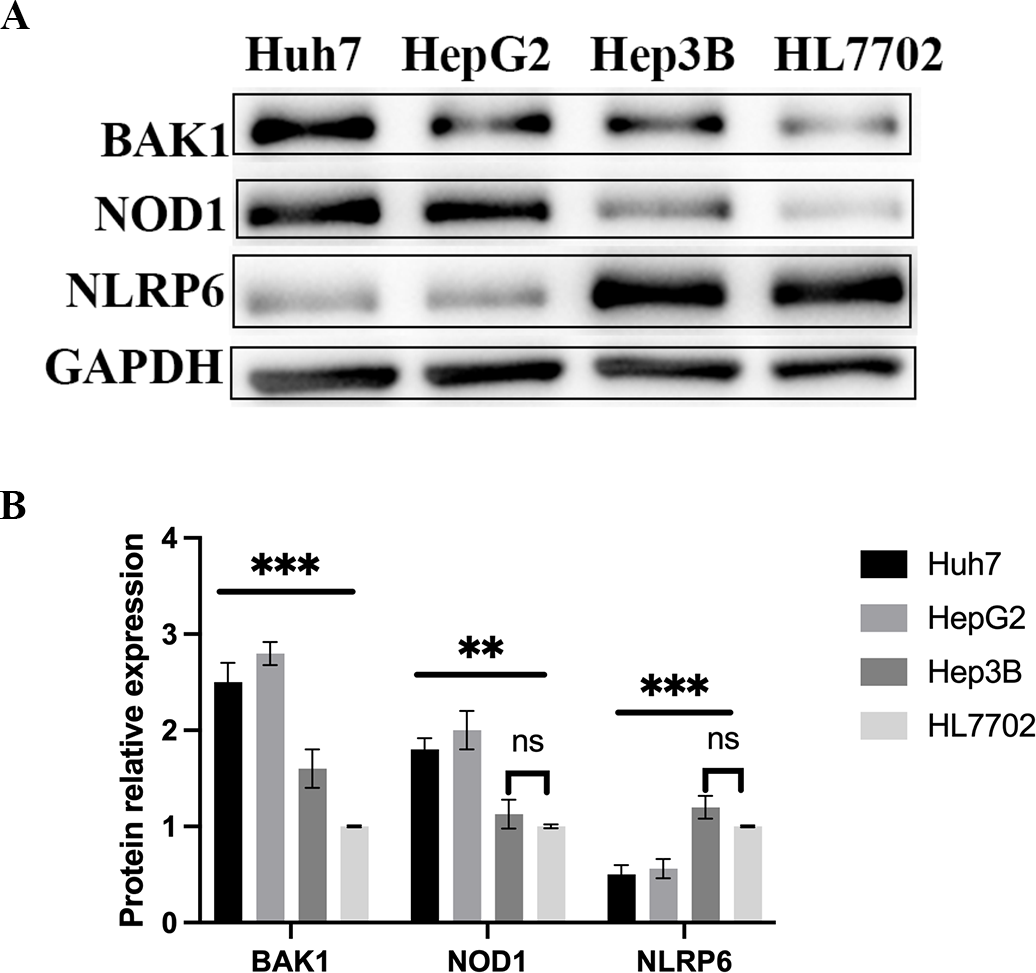

Supplement: Supplementary file 5 — Additional file 5. ( A)The protein expression of BAK1, NOD1 and NLRP6 in different cell lines by Western blot. ( B) The densitometric quantification levels of BAK1, NOD1 and NLRP6. **P < 0.01, ***P < 0.001, ns = no significant. [file 41065_2023_288_MOESM5_ESM.tif]
